# How wide is the cone of direct gaze?

**DOI:** 10.1098/rsos.180249

**Published:** 2018-08-01

**Authors:** Tarryn Balsdon, Colin W. G. Clifford

**Affiliations:** School of Psychology, UNSW Sydney, Australia

**Keywords:** gaze perception, social cognition, person perception

## Abstract

The cone of direct gaze refers to the range of gaze deviations an observer accepts as looking directly at them. Previous experiments have calculated the width of the cone of direct gaze using the gaze deviations actually presented to the observer, however, there is considerable evidence that observers actually perceive gaze to be systematically more deviated than actually presented. Here, we examine the width of the cone of direct gaze in units of perceived gaze deviation. In doing so, we are able to disambiguate differences in width both within and between observers that are due to differences in their perception of gaze and due to differences in what observers consider to be looking at them. We suggest that this line of inquiry can offer further insight into the perception of gaze direction, and how this perception may differ in clinical populations.

## 1. Introduction

The perception of being looked at is an important social signal that frequently signals another's intention to communicate with us [1,2]. A preference for direct gaze develops early, with newborn infants already showing a preference for faces with direct gaze over averted gaze [3] and by adulthood, a direct gaze advantage is shown across a number of tasks, such as in visual search tasks [4], in breaking from continuous flash suppression [5] and in the orienting of attention [6]. Despite this apparent specialization for detecting direct gaze, evidence suggests that observers will accept a wide range of gaze deviations as looking directly at them, spanning 8–9° in horizontal diameter [7]. This is especially large, considering performance thresholds in discriminating gaze deviations have been estimated to be less than 1 min of arc [8]. Accordingly, it has been suggested that observers may adopt some caution when deciding whether they are being looked at, to avoid the cost of missing direct gaze, which is greater than the cost of a false alarm, or characterizing averted gaze as direct [9].

**Figure 1:**
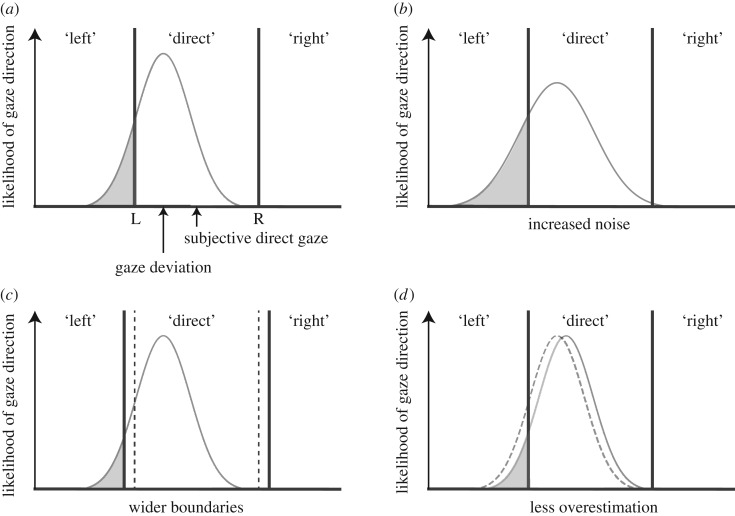
Psychophysical model of the cone of direct gaze, and transformations. (*a*) The cone of direct gaze model, where presented gaze deviations form a Gaussian probability distribution on an internal axis of gaze deviation. The boundaries L and R on the internal axis are used to decide whether to respond ‘left', ‘direct', or ‘right’. Here, the likelihood of the observer responding ‘left’ is represented by the grey area under the Gaussian distribution, the likelihood of responding ‘direct’ is the white area, and the observer will not respond ‘right' to this gaze deviation. (*b*) Increased noise (either external or internal noise) is represented as an increased standard deviation of the Gaussian distribution, creating more variable responses. (*c*) Wider category boundaries will allow the observer to accept a wider range of gaze deviations as looking directly at them. (*d*) Less overestimation, the observer perceives the gaze deviation as more direct and the distribution shifts towards subjective direct gaze. Although the category boundaries remain in the same location on the internal axis, they differ with respect to the actual presented gaze deviation.

The cone of direct gaze [7] describes the range of gaze deviations that an observer will accept as looking directly at them. The width of the cone of direct gaze is dictated by two factors: the perceived direction of gaze and the boundary the observer chooses at which point they no longer consider gaze to be directed at them. To measure the cone of direct gaze, Mareschal and co-workers [10] proposed a psychophysical model that parametrizes the factors influencing observers' judgements of whether gaze is direct, based on the assumptions that the observer is comparing noisy evidence on an internal axis of gaze deviation to two criteria (the boundaries of leftward versus direct gaze, and rightward versus direct gaze), as illustrated in figure 1*a*. This model, therefore, allows for the disambiguation of changes in behavioural responses based on changes in the sensory noise and changes in the observers' boundaries (for example, becoming more cautious and accepting perceptually greater gaze deviations as direct), as shown in figure 1*b,c*. Indeed, in comparing modelled responses to clear stimuli and stimuli where noise is added to the eye-region, decreasing the sensory evidence for gaze direction, Mareschal and co-workers [10] found the fitted parameters reflected an increase in the sensory noise when responding to noisy stimuli, as is consistent with increasing observers' uncertainty.

Previous measures of the cone of direct gaze have used the actual gaze deviations of the stimulus at the observer's response boundaries to calculate the range of gaze deviations the observer classifies as direct. This gives an indication of the range of gaze deviations presented to the observer that are classified as direct, but neglects the possibility that the perceived direction of gaze may systematically differ from the actual presented gaze deviation. This possibility has been demonstrated across a number of experiments where observers are asked to estimate the gaze direction of a stimulus, showing that observers consistently overestimate gaze deviations as more averted. By asking observers to indicate where on a scale gaze was directed, Anstis *et al*. [11] found the reported horizontal gaze deviation could be related to the actual presented gaze deviation by a line with a slope between 1.50 and 1.82 (i.e. an overestimation of gaze aversion of 50–82%). Similar measures have been obtained when asking observers to adjust a virtual ball with a pointer marked on it to be oriented in the same direction as gaze (slopes of 1.68 and 1.96; [12]). This overestimation seems unrelated to the motor response, as observers have also been found to overestimate in verbal reports of the angle of gaze deviation in schematic faces (with a slope of 1.35; [13]), and there is some evidence that overestimation occurs across three dimensions of gaze deviation [14,15]. The consistency of this overestimation bias across large differences in experimental stimuli and response parameters suggests that the overestimation is perceptual in nature, as opposed to a response bias where observers simply adopt the tendency to exaggerate their responses.

That perceived gaze direction differs systematically from the presented gaze direction causes two problems for making inferences about the width of the cone of direct gaze. First, the overestimation effect suggests that the range of gaze deviations the observer categorizes as direct corresponds to a wider range of perceived deviations than is reflected by the measurements based on the actual presented gaze deviations. Second, within and between observer variability in overestimation makes it difficult to understand the true cause of a difference in the calculated width of the cone of direct gaze. That is, the observer's category boundaries for direct gaze are based both on the perceived gaze direction and the cautiousness with which they categorize gaze as direct. So a difference in the width of the cone of direct gaze could be the result of a change in the perceived direction of gaze (for example, less overestimation, such that the same presented gaze deviations are represented along a less sparse internal evidence axis, while the boundaries stay in the same internal position on that axis, as shown in figure 1*d*) or a change in the observers' cautiousness (such that perceptually greater gaze deviations are accepted as direct, as shown in figure 1*c*).

This distinction is especially important for understanding differences in gaze perception that are observed in certain clinical populations. For example, patients with social anxiety disorder (SAD) have been shown to demonstrate a larger cone of direct gaze when making judgements in the presence of a second irrelevant face stimulus [16], and there is some evidence that this may be reduced after treatment with cognitive behavioural therapy [17]. In subclinical populations, a wider cone of direct gaze is measured in males high in social anxiety compared with those low in social anxiety [18], and other social factors have been shown to influence the width of the cone of direct gaze in the general population, such as the emotional expression of the face [19] and when the stimulus is accompanied by self-relevant auditory signals [20]. These changes to the cone of direct gaze could be the result of actually perceiving gaze differently, or changes in the category boundaries. Under the current framework, it is difficult to unequivocally conclude whether these more social changes to behavioural responses to gaze stimuli constitute a difference in the perception of gaze direction, or in the cautiousness with which observers make judgements of gaze stimuli, or some combination of these.

A similar problem is encountered when observers are presented with more uncertain sensory evidence. Mareschal *et al*. [21] have demonstrated that gaze is perceived as more direct with increasing uncertainty (when noise is added to the eye region). Other, more natural stimulus manipulations may also add uncertainty, such as presenting stimuli in the periphery [22,23] or presenting gaze in the context of different head orientations, which influences the perceived direction of gaze [24–27]. However, it may also be the case that observers also adopt a more cautious response strategy when confronted with uncertain sensory evidence. Without accounting for the perceived direction of gaze, it is unclear to what extent perceptual and decision-making factors contribute to the response strategy of observers in the face of uncertain evidence.

The current experiment seeks to measure the width of the cone of direct gaze using the observer's perceived gaze deviations as the unit for the width. A further objective is to examine the width of the cone of direct gaze under conditions of uncertainty. By using the perceived gaze deviation as the unit for the width of the cone of direct gaze, it is possible to test whether the wider cone of direct gaze measured under conditions of uncertainty is purely the result of perceiving gaze as more direct, or if it also results from increased cautiousness, where the observer accepts perceptually wider gaze deviations as directed at them. Observers were therefore asked to complete two tasks, an estimation task, where the perceived direction of gaze is computed against the presented gaze deviations, and a categorization task, where the cone of direct gaze model can be fitted to observers' responses of whether gaze was directed at them. Three levels of uncertainty were tested by adding sunglasses of differing opacity to the stimuli; completely clear (0% opacity), mid-dark glasses (99.7% opacity) and very dark glasses (99.9% opacity), where the sensory evidence for gaze direction was limited by the reduction in relative contrast within the eye region. The cone of direct gaze was then examined as has been computed previously, and by recalculating the width to account for the perceived gaze directions of the stimuli.

## 2. Methods

### 2.1. Participants

Twenty participants were recruited from the UNSW undergraduate research participation scheme and were given course credit for their participation. Prior to participating, participants gave written informed consent. Ethical approval for this study was granted by the UNSW Human Research ethics committee, which adheres to the declaration of Helsinki. All participants had normal or corrected to normal vision.

### 2.2. Apparatus

Stimuli were displayed on a 32' Display++ LCD monitor (Cambridge Research Systems, Rochester, UK) with a refresh rate of 120 Hz and resolution 1920 × 1080, with a grey background, mean luminance 60 cd m^−2^. Participants sat 57 cm from the screen with their chin on a chin rest.

### 2.3. Stimuli

One face stimulus was generated using FaceGen Modeller 3.5. The eyes were independently rotated according to precise coordinates in Blender 2.70, which was also used to add sunglasses to the face; the opacity of the sunglasses was also controlled. To control for any effect of asymmetry, the face was presented flipped along the vertical axis on half the trials. Face stimuli were presented such that the distance between the pupils was approximately 6.3 cm, which falls within the normal range of interpupillary distances for humans [27]. Stimulus presentation was controlled using Matlab and the Psychophysics Toolbox extensions [28–30]. Stimuli were presented for 400 ms, with a 100 ms raised cosine ramp temporally bordering 200 ms at full contrast. The position of the stimulus was jittered from trial to trial to be within 20 × 20 pixels (0.74 cm) of the screen centre.

### 2.4. Procedure

**Figure 2:**
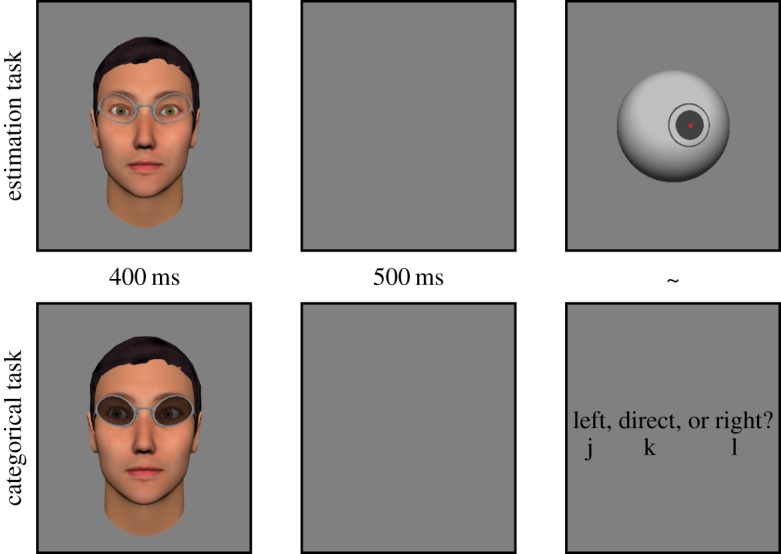
Trial procedure for each task. The top set shows an example estimation task trial, demonstrating 0% opacity sunglasses. The bottom set shows an example categorization task trial, illustrating the approximate moderate opacity (99.7%) sunglasses. Stimuli were the same across tasks. Response cues not to scale with stimuli.

Participants were asked to complete two tasks; an estimation task and a categorization task. The tasks were completed in separate blocks and the order of tasks was counterbalanced between participants. Before each task, participants followed step-by-step instructions and completed five practice trials with the experimenter present, to ensure that they understood how to respond. In the estimation task, observers were presented with a stimulus, followed after 500 ms by a ‘pointer', a virtual sphere that could be rotated to adjust the horizontal angle of the central target, by moving the computer mouse. Observers were asked to orient the pointer to be the same as the direction of gaze of the stimulus they just saw, and enter their response by clicking either of the mouse buttons. The initial orientation of the pointer was randomized on each trial. In the categorization task, observers saw the same set of stimuli, but this time were asked to decide if gaze was directed left, right, or directly at them. Responses were entered using the ‘j', ‘k' and ‘l' keys of a standard qwerty keyboard to indicate leftward, direct and rightward gaze, respectively. Observers were reminded of this response contingency with text instructions presented on the screen 500 ms after each stimulus, and this also served as a cue to respond. Example trials for each task are presented in figure 2. The direction of gaze of the stimulus was varied between −12° and 12° in steps of 3° and there were three levels of sunglasses opacity; 0% (clear) 99.7% (dark) and 99.9% (almost opaque). Observers made 16 responses to each gaze direction in each level of opacity, giving a total of 432 trials per task, and the two tasks were completed in less than half an hour.

### 2.5. Analysis

In the estimation task, a slope and intercept were fit to the median pointer response at each gaze deviation, for each opacity level separately, using the method of least squares. The intercept of the line marks perceptually direct gaze, while the slope describes the relationship between veridical and reported gaze direction. The variability of responses around the median response is indicative of both perceptual and motor noise.

In the categorization task, the proportion of left, right and direct responses at each gaze deviation were fit with a model to describe the cone of direct gaze, as presented in [10], using the method of least squares. The model involves three parameters: the position of the centre of the cone of direct gaze (or perceptually most direct gaze), the width of the cone, and standard deviation of the Gaussian sensory noise. The model assumes that the decision is made based on the position of a noisy signal relative to the category boundaries on an internal axis of gaze direction, such that if the signal + noise is left of the left-most boundary, the observer chooses ‘left', if it is between the two boundaries the observer chooses ‘direct', and if it is to the right the observer chooses ‘right'. This model was fit separately for each level of opacity.

Individual responses, summary statistics, and fitted lines were visually inspected for each observer. At this point, two observers were rejected from further analysis as their responses did not vary with gaze deviation. In the estimation task, no other observer responded in a way that differed markedly from linear, and similarly in the categorization task, the assumption of Gaussian noise did not appear unjustified for the observers included in the analysis, with the model accounting for 97.21% of the variance in the data, on average.

**Figure 3:**
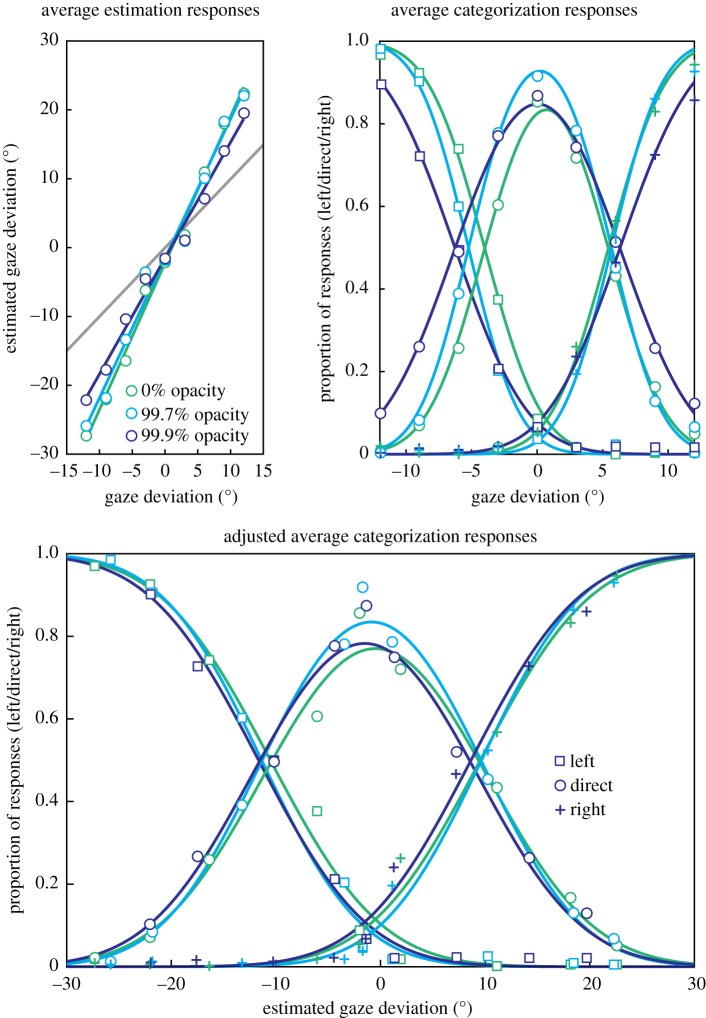
Average responses in the estimation and categorization tasks. Solid lines show the implemented analysis described above, fit to the actual data points shown in markers. The grey line in the estimation graph indicates veridical responding (a slope of 1), an increased slope reflects the overestimation effect. The adjusted average plots the categorization responses against the estimated gaze deviation from the estimation task. The widths of the *x*-axes of the two categorization graphs equate perceived and actual gaze deviations; however, the *x*-axis of the estimation responses is relatively contracted.

The cone of direct gaze model was then fit to each observer's categorization responses again, but instead of describing the gaze deviation they were responding to with the veridical gaze position, their median pointer response was used. By way of demonstration, if the observer had responded 80% of the time that a 9° gaze deviation was rightward, but in the estimation task they had indicated that 9° of gaze deviation appeared deviated by 14°, then the model was now fit based on responding 80% of the time that a gaze deviation of 14° was rightward. The parameters here thus correspond to the perceived direction of gaze that is most direct, the width of perceived gaze deviations that are considered direct, and the standard deviation of sensory noise on this perceptual scale, assuming that responses in the estimation task accurately reflect perceived gaze direction. The implementation of this analysis, averaged across observers, is shown in figure 3.

## 3. Results

**Figure 4:**
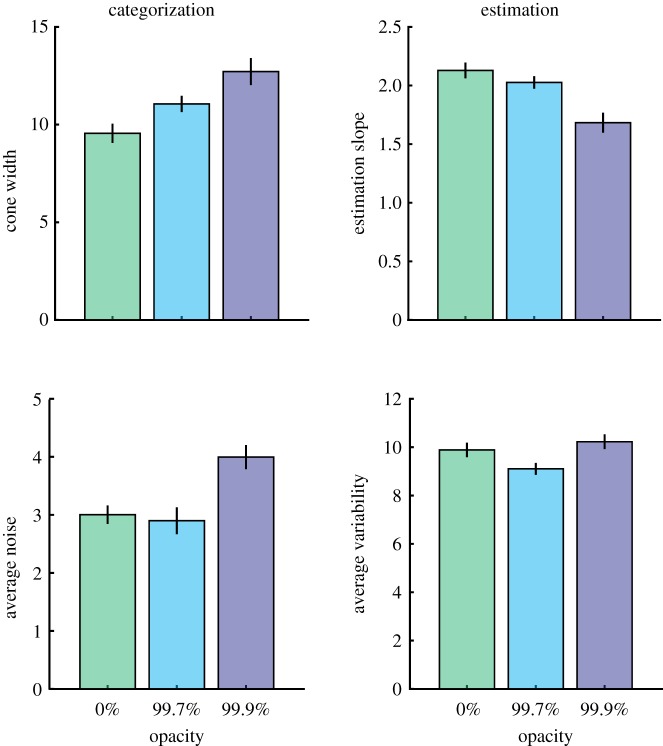
The effect of opacity on measures in the categorization and estimation tasks. Error bars show 95% within subjects confidence intervals.

It was expected that increasing the sunglasses' opacity would (i) increase the noise associated with participants' responses (measured by the noise parameter in the categorization task and the variability in responses around the median response in the estimation task), (ii) increase the width of the cone of direct gaze in the categorization task, and (iii) decrease the slope of the estimation judgements. This was tested using four separate repeated measures ANOVAs, where the *p*-values have been corrected to include a Bonferroni correction for multiple comparisons, and a Greenhouse–Geiser correction has been applied where violations of sphericity occur. In the categorization task, opacity was found to significantly increase the width of the cone of direct gaze (*F*_2,23.2_ = 22.67, *p *< 0.001, $\eta_{p}^{2}=0.586$) and increase noise (*F*_2,32_ = 24.27, *p* < 0.001, $\eta_{p}^{2}=0.603$). In the estimation task, increasing opacity significantly decreased the slope relating perceived and actual gaze direction (*F*_2,32_ = 30.225, *p *< 0.001, $\eta_{p}^{2}=0.654$) and had a significant effect on the variability in responses (*F*_2,32 _= 10.928, *p *< 0.001, $\eta_{p}^{2}=0.406$). Follow-up paired *t*-tests showed this pattern in response variability was not quite as predicted, with the difference being driven by reduced variability in the moderate opacity condition, with no significant difference between the most opaque condition and the zero opacity condition (*M*_0–99.7_ = 0.78°, *t*_16_ = 3.45, *p* = 0.003; *M*_99.7–99.9_ = −1.12°, *t*_16_ = −4.84, *p *< 0.001; *M*_0–99.9_ = −0.34°, *t*_16_ = −1.22, *p* = 0.24). Average measurements are shown in figure 4.

**Figure 5:**
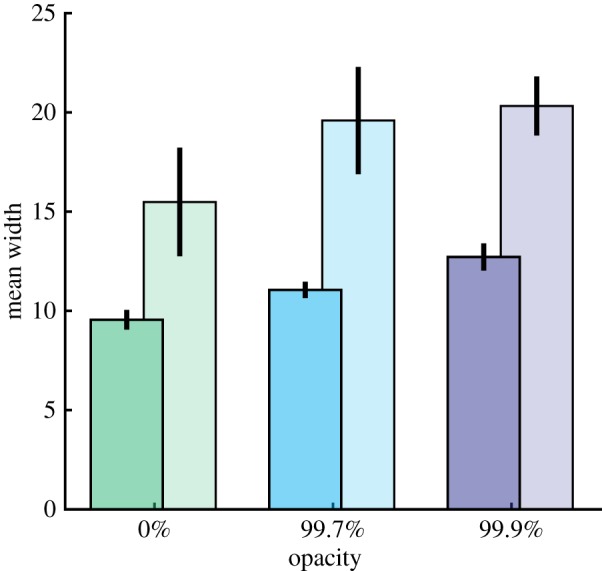
Width of the cone of direct gaze measured with actual gaze deviation and estimated gaze deviation. Widths (in degrees) based on estimated gaze deviation (from the estimation task) are the lighter, larger bars. Error bars show the 95% within subject confidence intervals.

Finally, the cone of perceived direct gaze was measured by refitting the gaze cone model to the data adjusted such that responses corresponded to perceived gaze deviation (as measured in the estimation task) rather than actual gaze deviation. The average cone widths are presented in figure 5. A repeated measures ANOVA with measure (veridical versus perceived) and opacity (0, 99.7 and 99.9%) as within-subjects factors, revealed a significant effect of opacity (*F*_2,32_ = 7.133, *p* = 0.003, $\eta_{p}^{2}=0.308$) and a significant effect of measure (*F*_2,31_ = 20.225, *p* < 0.001, $\eta_{p}^{2}=0.558$) but no significant interaction (*F*_1.3,20.3_ = 0.837, *p* = 0.398, $\eta_{p}^{2}=0.05$), indicating that the adjustment significantly increased the measures of the cone of direct gaze, and the same effect of opacity was demonstrated. To check that increased opacity still corresponded to significantly increased width of the cone of direct gaze in the adjusted measure, a *post hoc* paired samples *t*-test was used to compare the width in the 0% and 99.9% opacity conditions, this revealed a significant result (*t*_16_ = 2.794, *p* = 0.013 uncorrected).

## 4. Discussion

This experiment examined measures of perceived gaze direction across two tasks, under different levels of uncertainty. Measurements across both tasks were on average very similar to previous experiments, demonstrating a wide cone of direct gaze in the categorization task (on average, 9.55° in the 0% opacity condition) and a systematic overestimation of gaze deviation in the estimation task (with an average slope of 2.13 in the 0% opacity condition). In both the categorization and the estimation tasks, increased uncertainty biased observers to report gaze as more direct, as has been shown in previous experiments [21,31].

**Figure 6:**
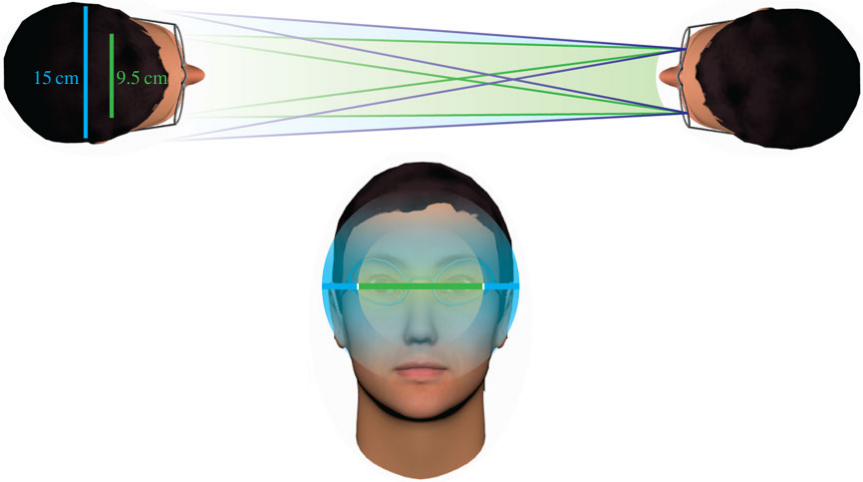
Cone of direct gaze relative to the observer. The calculated values are based on the average cone widths, taking into account the viewing distance of the observer (57 cm). The green cone shows the width measured with the actual deviations presented to the observer, the blue cone shows the width measured by the perceived deviation, based on the estimation responses. The vertical extent of the cone of direct gaze has not been measured here, although there is some evidence that the vertical extent may be similar to the horizontal extent [33] and overestimation has also been demonstrated for vertical gaze deviations [21].

The width of the cone of direct gaze was recalculated based on each observer's perceived directions of gaze at each presented gaze deviation. This resulted in a significantly increased width compared with when the presented gaze deviations were used as the unit. On average, the width of the cone of direct gaze adjusted for perceived gaze direction, corresponded to 15 cm, which encompasses the average approximate breadth of the human head (15 cm, [32]). The difference in these measures is shown in figure 5 and illustrated in figure 6. This wider cone of perceived direct gaze suggests that observers may be taking into account the extent of their own heads when considering whether gaze is direct, or that they are interpreting the task as whether gaze is directed in the vicinity of their own face. However, it is also unclear whether changing the instructions to specify mutual gaze, for example, would decrease the width of the cone of direct gaze: the unadjusted measurements in the 0 opacity condition were in line with previous measurements made when observers were asked to adjust the gaze of virtual heads to be looking directly into their eyes [7]. The width of the cone may therefore be due to genuine perceptual variability coupled with the cost of missing direct gaze. Although there is little evidence for qualitative differences between measurements made using real human faces and the controlled stimuli such as those used here [7], it may be interesting to test how these factors influence the measurements of the cone of the direct gaze in real human faces also.

A further aim of the experiment was to test the effect of uncertainty across both judgements. In the categorization task, uncertainty was also shown to increase the measured noise in observers' responses. However, in the estimation task, this pattern was not observed. Rather, the moderate uncertainty condition (99.7% opacity) was shown to induce significantly reduced variability in estimation judgements compared with the other two conditions. This difference from the prediction may have been the result of increased variability in the low uncertainty condition, where observers may have been less cautious in responding when the decision was easier. Furthermore, the variability in the estimation task was probably dominated by ‘motor' rather than ‘sensory' factors, as evidenced by the increased magnitude of variability in the estimation task compared to the noise measured in the categorization task.

The examination of both overestimation *and* the width of the cone of direct gaze within participants also reveals more about the effect of uncertainty on the perception of gaze direction. When we adjusted the measures of the width of the cone of direct gaze to account for the overestimation in perceived gaze direction across levels of uncertainty, there is still an increased width of the cone of direct gaze with increased uncertainty. This suggests that observers are both perceiving gaze as more direct under uncertainty, and adjusting their category boundaries to accept perceptually greater gaze deviations as directed at them.

In conclusion, these experimental results suggest that observers set their category boundaries for direct gaze to approximately account for the perceived extent of their own heads. When uncertain sensory information is provided, observers not only perceive gaze to be more directed at themselves, they also become more cautious, by accepting perceptually greater gaze deviations as directed at themselves. By accounting for perceived gaze deviation when measuring the cone of direct gaze, far more could be understood about the way in which observers were judging gaze stimuli. Therefore, this method would provide greater insight in other cases, such as in measuring the width of the cone of direct gaze in clinical populations, such as people with SAD, or people with autism spectrum disorder.

## Supplementary Material

supplementary data

## Data accessibility

Data have been attached as electronic supplementary material.
